# A comparative study of DistilBERT and Perceiver for deceptive review detection in hospitality review data

**DOI:** 10.3389/fdata.2026.1918383

**Published:** 2026-09-09

**Authors:** Ioana Ximena Remeş, Felicia Mirabela Costea, Cornelia Aurora Gyorödi

**Affiliations:** Department of Computers and Information Technology, Faculty of Electrical Engineering and Information Technology, University of Oradea, Oradea, Romania

**Keywords:** deceptive review detection, DistilBERT, hospitality review analysis, natural language processing, Perceiver, transformer models

## Abstract

Deceptive reviews on hospitality platforms can undermine consumer trust and affect the reliability of online reputation systems. This study presents a comparative empirical evaluation of two deep learning pipelines for deceptive review detection: a DistilBERT-based pipeline fine-tuned end-to-end on review text and complemented with rating and sentiment features, and a Perceiver-based pipeline operating on fixed sentence embeddings generated by a frozen encoder. The experiments were conducted on a combined English-language corpus of 3,322 hotel reviews assembled from the Myleott benchmark corpus and a Kaggle dataset of authentic Ritz-Carlton New York reviews, comprising approximately 2,520 genuine and 800 deceptive reviews. The corpus was evaluated using stratified 80/20 train–test splits, with 2,657 reviews used for training and 665 for testing in each split, and the deep learning models were trained for 10 epochs. To assess the robustness of the findings, the evaluation was repeated across three independent random seeds and compared with majority-class and TF-IDF plus logistic regression baselines. DistilBERT achieved the most stable performance, reaching an average accuracy of 0.9348 ± 0.0014, an F1-score of 0.9569 ± 0.0013, and a Matthews correlation coefficient of 0.8240 ± 0.0012. In contrast, the original Perceiver pipeline showed a strong tendency to collapse toward the majority class, which limited its ability to identify deceptive reviews despite apparently high recall. Although class-weighted training improved Perceiver's behavior, its results remained less reliable and more variable than those obtained with DistilBERT. The balanced TF-IDF plus logistic regression baseline performed close to DistilBERT, indicating that well-configured traditional natural language processing methods remain competitive on datasets of this size. The ablation analysis further indicates that the rating and sentiment features contribute primarily to the stability of the model across different train–test splits, rather than providing a substantial independent gain in discriminative performance. The results indicate that the fine-tuned DistilBERT pipeline provides the most robust option in the evaluated setting, while also emphasizing the importance of reporting corpus composition, test-set size, class imbalance, and imbalance-aware metrics when developing deceptive review detection systems for hospitality review data.

## 1 Introduction

Against the backdrop of rapid technological advancement, online reviews have emerged as one of the most influential sources of information in consumer decision-making. Sectors such as tourism and hospitality are particularly susceptible to the effects of publicly expressed opinions, which have increasingly come to function as decisive factors in service and destination selection. This growing influence, however, has been accompanied by a concerning phenomenon: the proliferation of fraudulent reviews.

Such reviews, deliberately crafted to mislead readers, can produce significant adverse effects–either by artificially inflating the perceived quality of inferior services or by systematically disparaging competitors–thereby generating unjustified competitive advantages or undermining the reputation of legitimate businesses. Consequently, the automated detection of fraudulent reviews represents a critical challenge, both for safeguarding end users and for preserving the integrity of publicly available information.

In an environment where online platforms facilitate the rapid publication and widespread dissemination of user-generated content, deceptive reviews can no longer be regarded as an anomaly, but rather as a pervasive and frequently encountered reality. This phenomenon not only distorts individual user decisions but also poses a broader threat to public trust in online evaluation systems.

Artificial intelligence provides a range of methodological tools that can be used to address this challenge. Unliske traditional approaches based primarily on pre-defined grammatical or lexical rules, recent deep learning methods are able to capture more complex patterns in textual data. Their analysis can also be complemented by structured information, including numerical ratings, the frequency of reviews, and other relevant metadata. In addition, information from different sources can be considered within the same analytical framework. Images, for example, may provide further contextual evidence alongside the written review. This combination makes AI-based methods particularly relevant to the identification of potentially deceptive reviews.

The use of artificial intelligence has also expanded considerably within the hospitality industry. Hotels increasingly rely on AI-based applications to improve operational processes, manage interactions with customers, and support decisions based on available data. These technologies may contribute to more personalized guest experiences and can assist organizations in processing the large amount of feedback generated through online platforms. In the specific case of English-language hotel reviews, AI methods offer a practical means of examining customer feedback at scale. This is particularly useful for deceptive review detection, since machine learning models can identify recurring linguistic and behavioral characteristics in review data and assess whether such characteristics differ between authentic and deceptive content.

Against this background, the present study investigates the use of automated methods for detecting deceptive reviews in the hospitality domain. The issue is relevant not only from a methodological perspective, but also because the reliability of online rating and review systems depends, to a considerable extent, on the authenticity of the content they contain. Misleading reviews may affect consumer choices and create an inaccurate representation of a hotel or service. Detecting such content can therefore contribute to maintaining greater transparency and reliability in digital review environments. With this objective in mind, the study examines two advanced artificial intelligence models and compares their effectiveness in distinguishing deceptive hotel reviews from authentic ones. The present paper therefore investigates the performance of artificial intelligence models for deceptive review detection in the hospitality sector through a comparative analysis of DistilBERT and Perceiver. The comparison focuses on two distinct architectural paradigms: DistilBERT, a pre-trained Transformer-based language model designed for efficient natural language understanding, and Perceiver, a general-purpose architecture that relies on latent cross-attention to process inputs of different modalities and scales. By examining these models under a common dataset and evaluation protocol, the study aims to provide a clearer understanding of their strengths, limitations, and practical suitability for this classification task.

This study aims to detect deceptive reviews by providing a comparative evaluation of two distinct attention-based deep learning pipelines within the hospitality domain. More specifically, it compares DistilBERT, a distilled Transformer model fine-tuned end-to-end on review text, with a Perceiver-type latent cross-attention model operating on fixed sentence embeddings generated by a frozen encoder. Given these different learning and input conditions, the study considers the two approaches as complete processing pipelines rather than as directly comparable architectures, treating this asymmetry as an important limitation when interpreting the results. The study further highlights the importance of evaluating model performance beyond conventional aggregate indicators, particularly in the presence of class imbalance. The perfect recall obtained by the original Perceiver pipeline illustrates this issue, as it results from the systematic prediction of the majority class rather than from an effective ability to distinguish deceptive content. To provide a more reliable assessment, the analysis therefore incorporates imbalance-aware measures, including specificity, balanced accuracy, and the Matthews correlation coefficient, alongside conventional performance metrics. All results are reported as mean ± standard deviation across three independent stratified train–test splits to provide a more robust estimate of model performance. In addition, the two deep learning approaches are evaluated against a majority-class dummy classifier and a TF-IDF with Logistic Regression baseline trained directly on the raw review text, providing reference points for assessing their relative performance. An ablation analysis is also conducted to examine the contribution of auxiliary numerical features, while a class-weighted variant is used to investigate whether the majority-class-collapse behavior observed in the original Perceiver pipeline can be mitigated through a standard class-imbalance correction strategy. Finally, the study identifies and discusses the limitations of the evaluated approaches, particularly the instability and majority-class collapse observed in the Perceiver pipeline, while outlining directions for developing more robust deceptive review detection systems.

## 2 Related work

Online reviews play an important role in the way consumers choose accommodation in the hospitality sector. Recent studies suggest that more than 90% of users read reviews on platforms such as TripAdvisor, Booking.com, and Google Reviews before making a reservation (Nain, 2025). These reviews can shape how potential guests perceive a hotel and may influence their final booking decision. Negative feedback, particularly when it concerns recurring problems, can damage a hotel's reputation, whereas positive reviews can strengthen customer confidence and contribute to higher occupancy. Since hospitality services are closely associated with trust and the quality of the guest experience, the credibility of online feedback is therefore important for both hotel reputation and long-term relationships with customers.

The earliest forms of online reviews emerged during the 1990s and 2000s, coinciding with the rapid expansion of electronic commerce. Platforms such as Amazon, Yelp, and TripAdvisor played a pivotal role in institutionalizing open consumer feedback. Amazon was among the first e-commerce platforms to implement a public product review system (Dellarocas, 2003), enabling users to rate and comment on their purchasing experiences, thereby directly influencing the consumption decisions of other users. Yelp became widely recognized for its detailed evaluations of restaurants and local businesses (Finance, 2024), while TripAdvisor established itself as a leading platform for hotel and travel-related reviews (Tripadvisor, 2025). These platforms are particularly relevant to the present study given their high usage rates, global reach, and demonstrable influence on consumer decision-making.

Among the most prominent applications of AI in the hospitality industry, conversational agents provide continuous customer support, handling reservations and responding to frequently asked questions, thereby reducing staff workload and improving response consistency (Vinnakota, 2023). Additionally, machine learning algorithms enable the generation of personalized recommendations based on customer behavior, preferences, and interaction history, contributing to enhanced satisfaction and loyalty (Gatera, 2024).

Natural language processing (NLP) techniques are widely employed for the automated analysis of online reviews, facilitating sentiment extraction and the identification of recurrently mentioned aspects, which in turn support managerial decision-making (Birim et al., 2024). AI is further applied in dynamic pricing strategies, whereby room rates are adjusted in real time according to demand fluctuations, seasonality, and competitive conditions, thereby optimizing revenue management and profitability (Talón-Ballestero et al., 2022). Predictive models additionally support demand forecasting and resource planning, enabling more efficient allocation of staff and operational resources while reducing costs (Wu et al., 2020).

The transformative impact of digital technologies on the hospitality industry has been examined in Nain (2025), with particular emphasis on the central role of online reviews and ratings in shaping consumer behavior and business performance. The author argues that user-generated content–particularly in the form of electronic word-of-mouth–significantly influences customer trust, booking intentions, and brand competitiveness.

In Mazin et al. (2024), the authors investigate the effectiveness of Transformer-based deep learning models in detecting deceptive online reviews, underscoring the growing relevance of this issue in the context of e-commerce expansion. The study presents a comparative analysis of several architectures, including BERT, RoBERTa, and XLNet, as well as deep learning models such as BiLSTM and CNN. The findings demonstrate that Transformer-based models achieve superior performance in distinguishing between genuine and fraudulent reviews. In particular, RoBERTa attains the highest classification accuracy (97.1%), albeit at the cost of greater computational resources.

Recent research has also addressed the detection of AI-generated or paraphrased reviews, particularly those produced by advanced generative language models such as ChatGPT. In AlQadi et al. (2025), the authors propose an explainable deep learning framework based on a fine-tuned DistilBERT model, augmented with linguistic pre-processing techniques including part-of-speech tagging and lemmatization. The study further incorporates Explainable Artificial Intelligence (XAI) methods–specifically LIME and SHAP–to enhance model transparency and interpretability. The experimental results demonstrate strong predictive performance, with an accuracy of 97.25% and an F1-score of 97.56%, confirming the suitability of Transformer-based architectures for detecting AI-paraphrased deceptive content.

Yadav et al. (2024) propose a novel approach to fake consumer review classification based on a Chi-Squared Text Convolutional Neural Network (TCNN), designed to address the limitations of existing methods that rely on low-discriminative features and yield suboptimal accuracy. The proposed framework integrates text pre-processing (tokenization and stemming), feature extraction via Part-of-Speech (POS) tagging and Bag-of-Words representation, and feature selection through the chi-squared statistical test. Final classification is performed using a TCNN architecture, achieving an accuracy of 98.45% on the Yelp dataset.

Although previous work has shown that deep learning, and particularly Transformer-type architecture, can achieve very high levels of accuracy in distinguishing between genuine reviews and fraudulent or AI-paraphrased ones, a significant portion of these results are obtained on relatively balanced datasets and under conditions that do not always reflect the constraints of real hotel review platforms.

In this context, the present study investigates the detection of deceptive reviews in the hospitality domain by comparing two deep learning approaches: DistilBERT, a distilled Transformer-based language model, and Perceiver, an architecture based on cross-attention mechanisms. Both models are evaluated on the task of classifying hotel reviews as either genuine or deceptive. Rather than focusing exclusively on overall classification performance, the analysis also considers how the models behave when the data are imbalanced and when computational resources are limited. Particular attention is given to the types of errors produced by each model, as these can reveal limitations that may not be apparent from standard performance metrics alone. Through this comparison, the study aims to provide a more detailed understanding of the strengths and limitations of the two approaches and to examine their suitability for developing reliable automated methods for deceptive review detection.

## 3 Features of DistilBERT and Perceiver

### 3.1 DistilBERT

DistilBERT is a Transformer-based architecture for natural language processing developed by Hugging Face, derived from the Bidirectional Encoder Representations from Transformers (BERT) model (Sanh et al., 2019). It is designed to reduce model size and computational complexity while preserving a high level of performance. Compared to BERT-base, DistilBERT retains approximately 40% fewer parameters and achieves faster inference, making it suitable for resource-constrained environments (Gandhi et al., 2025).

The model is obtained through a knowledge distillation procedure, where a pre-trained BERT model acts as a teacher and guides the training of a smaller student model. During this process, DistilBERT is trained to match the softened output probabilities (logits) of the teacher model, while also leveraging the original training objectives. The distillation loss typically combines multiple components, such as the Kullback–Leibler divergence between the teacher and student output distributions, masked language modeling loss, and cosine embedding loss between hidden states, enabling effective transfer of both predictive behavior and internal representations (Sanh et al., 2019).

Architecturally, DistilBERT preserves the core Transformer encoder design but reduces the number of layers (e.g., from 12 to 6 in the base configuration) and removes certain components such as token-type embeddings. Parameter sharing and initialization strategies derived from the teacher model contribute to faster convergence and improved generalization.

DistilBERT offers faster inference speed, lower computational cost, and competitive performance across a variety of natural language processing tasks, representing a favorable trade-off between efficiency and performance (Riyadi et al., 2024). Through its optimized architecture and extensive support in modern libraries, the model is well-suited for tasks such as review classification, semantic analysis, and deceptive content detection, which are relevant to the objectives of this study (AlQadi et al., 2025).

### 3.2 Perceiver

The Perceiver model, introduced by Jaegle et al. (2021a), represents a significant advancement of the Transformer architecture, designed to efficiently handle multimodal data such as text, images, audio, and tabular data, without requiring a specific input structure.

A defining feature of the Perceiver architecture is the introduction of a fixed-size latent array, which serves as an intermediate representation (Jaegle et al., 2021b). Instead of applying self-attention directly over the input tokens, the model first performs cross-attention between the input and the latent space, projecting the input into a smaller set of latent variables. Subsequent processing is performed entirely within this latent space using self-attention layers, significantly reducing computational complexity while preserving the ability to model long-range dependencies.

The architecture can process various types of input data–including text, images, audio, and structured data–without requiring substantial architectural changes (Jaegle et al., 2021a). This flexibility is achieved by treating inputs as generic arrays and applying shared attention mechanisms, making the model suitable for multimodal learning tasks.

From a scalability perspective, Perceiver allows efficient handling of large inputs by maintaining a constant-size latent representation, regardless of input length or resolution. This results in improved memory efficiency and enables the model to scale to tasks involving high-dimensional data that are impractical for conventional Transformer architectures.

The Perceiver model proposes an elegant and versatile architecture, well-suited for machine learning problems involving complex, heterogeneous, or large-scale data (Jaegle et al., 2021b). Within the scope of this study, Perceiver is evaluated in depth to assess its performance in deceptive review detection.

## 4 Methodology

### 4.1 Experimental environment

The experimental framework of this study was implemented using the Google Colab environment, selected for its cloud-based execution capabilities, integrated GPU support, and compatibility with contemporary machine learning libraries. The implementation was developed in Python 3, and all stages of the pipeline–data acquisition, pre-processing, model training, validation, and evaluation–were conducted within this environment.

### 4.2 Dataset construction

To ensure a robust comparative evaluation, the study employed two complementary datasets originating from distinct sources, with an initial number of 3,500 English-language hotel reviews. Following pre-processing, language filtering, and deduplication, the final corpus consisted of 3,322 English-language hotel reviews, partitioned under stratified 80%−20% train–test splits, yielding 2,657 training samples and 665 test samples per split. To assess the stability of the results, three independent stratified splits were generated with different random seeds (42, 123, and 2,024). The stratified split ensured that class proportions were preserved in both subsets.

The first dataset was obtained from the publicly available Myleott corpus and contains approximately 1,600 hotel reviews collected from 20 hotels located in Chicago. The dataset is balanced with respect to class distribution, comprising roughly 800 truthful and 800 deceptive reviews. It is organized hierarchically according to sentiment polarity (positive or negative), veracity (truthful or deceptive), and source category (TripAdvisor, MTurk, and Web). This corpus was introduced by Ott et al. (2011) and is publicly available at the author's repository.

The second dataset was sourced from Kaggle and contains approximately 1,900 hotel reviews in English, all written about a single hotel–the Ritz-Carlton, New York–and originally collected from Tripadvisor (2025). All reviews in this dataset were labeled as truthful, as they originate from a verified public source. Sentiment was derived from the rating field: reviews with a score of 3.5 or above were labeled positive, and those below that threshold were labeled negative. The dataset is publicly available on Kaggle (2024).

After merging the two datasets and applying pre-processing steps (language filtering, deduplication, removal of reviews shorter than 20 characters), the combined corpus contained approximately 2,520 genuine and 800 deceptive reviews, reflecting the imbalanced nature introduced by the entirely truthful Kaggle dataset. This class imbalance is relevant to the interpretation of the Perceiver model's behavior, as discussed in Section 6. In all reported classification metrics, the positive class corresponds to the majority (genuine/truthful) class, consistent with the label encoding used during training; detection of the minority deceptive class is therefore best assessed through specificity and the imbalance-robust metrics reported in Section 6.3 and 6.4.

### 4.3 Pre-processing pipeline

Before model training, the textual data were subjected to a standardized Pre-processing pipeline. This included text normalization and removal of irrelevant symbols, automatic language identification with retention of English-language reviews only, deduplication, exclusion of reviews shorter than 20 characters, and finally tokenization and transformation into numerical representations compatible with Transformer-based architectures.

### 4.4 Model training and evaluation

Two deep learning architectures were comparatively evaluated: DistilBERT (Sanh et al., 2019) and Perceiver (Jaegle et al., 2021a). DistilBERT was selected due to its computational efficiency and strong contextual language representation capabilities, while Perceiver was included because of its scalable latent attention mechanism and and its flexibility in processing heterogeneous inputs. Both models were trained under the same train-test protocol and evaluated on the same dataset to ensure methodological consistency.

To place these results in context and to test their stability, a second evaluation campaign was subsequently carried out. It comprised four reference configurations–a majority-class dummy classifier; TF-IDF features combined with logistic regression trained on the raw review text; the same pipeline with balanced class weights; and a Perceiver variant trained with a class-weighted binary cross-entropy loss (positive-class weight ≈ 3.15)–together with an ablation in which DistilBERT was retrained on the review text alone. The classical baselines were implemented in scikit-learn and trained on the same reviews, so that, unlike the logistic-regression reference reported in Table 1, they had full access to the original words rather than to frozen embeddings.

**Table 1:** Perceiver vs. Logistic Regression on frozen embeddings.

Metric (epoch 10)	Perceiver	Logistic regression
Accuracy	0.7594	0.7710
F1-score	0.8632	0.8700
Balanced accuracy	0.5000	0.5140

Every configuration in this campaign, including the two deep models, was evaluated on three independent stratified 80/20 train–test splits generated with the random seeds 42, 123, and 2,024, each preserving the class proportions of the corpus and a test set of 665 reviews (505 genuine and 160 deceptive). Results are reported as mean ± standard deviation across the three splits in Sections 6.4 and 6.5. Because the campaign involved a fresh training run for every model, its DistilBERT and Perceiver figures differ slightly from the single-run values reported in Sections 5–7; the discrepancy is small and is itself informative about the run-to-run variability of the training procedure.

The experimental evaluation was conducted over 10 training epochs. Model performance was assessed using nine classification metrics: accuracy, precision, recall, F1-score, specificity, balanced accuracy, the Matthews correlation coefficient (MCC), log loss, and ROC-AUC. These metrics were selected to provide a comprehensive evaluation of predictive performance, class discrimination capability, confidence calibration, and classification robustness. Representative checkpoints at epochs 3, 5, and 10 were selected for detailed comparative analysis, reflecting early convergence, intermediate adaptation, and final optimization behavior. The epoch-wise analyses in Sections 5–7 refer to this initial configuration.

### 4.5 Model architectures and input representations

The two models were applied through deliberately different input pipelines, dictated by their respective designs. For DistilBERT, review text was tokenized with the pre-trained DistilBertTokenizer (with padding and truncation) and passed through the DistilBertModel backbone. The pooled [CLS] representation was then concatenated with the two auxiliary structured features—the numerical rating and a binary sentiment indicator—and passed through a dropout layer and a small feed-forward head (two dense layers with ReLU activation and a final sigmoid). This custom model, denoted BERTWithFeatures, was fine-tuned end-to-end for 10 epochs using the AdamW optimizer and a binary cross-entropy objective (BCEWithLogitsLoss), with a batch size of 16. The integration of rating and sentiment is thus a late-fusion mechanism: the textual representation learned by the Transformer is combined with the structured features immediately before the classification head.

To quantify the contribution of the auxiliary structured features, an additional ablation experiment was conducted using a text-only DistilBERT configuration. In this variant, the pooled [CLS] representation was passed directly to the classification head without concatenating the numerical rating or the binary sentiment indicator. All remaining training settings, optimizer parameters, and evaluation protocol were kept identical to those of the full DistilBERT pipeline, allowing the influence of the auxiliary features to be assessed in isolation.

For Perceiver, the text was not tokenized or processed as a sequence. Instead, each review was first encoded into a single 384-dimensional vector using a frozen, pre-trained sentence-transformer (all-MiniLM-L6-v2), to which the rating and sentiment values were concatenated, yielding a 386-dimensional input. This fixed-length vector was then classified by a compact Perceiver configuration (32 latent vectors, latent dimension 64, depth 2, one cross-attention head and one self-attention head, cross-attention dimension 64, and Fourier positional encoding with a maximum frequency of 10), trained from scratch for 10 epochs with the Adam optimizer and a binary cross-entropy loss (BCELoss), with a batch size of 16. Two consequences of this design are important for interpreting the results (Sections 6 and 8): the text encoder is frozen and therefore not adapted to the deception-detection task, and the Perceiver operates on a single pooled sentence embedding rather than on a token sequence, so its latent cross-attention mechanism–intended to model structured or high-dimensional inputs–has little sequential structure to exploit in this setting.

To investigate whether the observed collapse of the Perceiver model toward the majority class was primarily caused by class imbalance, an additional experiment was conducted using a class-weighted binary cross-entropy loss. The positive-class weight was computed from the training data according to the class distribution (pos_weight ≈ 3.15), increasing the contribution of deceptive reviews during optimization while leaving the remaining experimental settings unchanged. The resulting model is referred to as Perceiver + class-weighted loss and is evaluated together with the original Perceiver configuration in Section 6.4.

**Figure 1:**
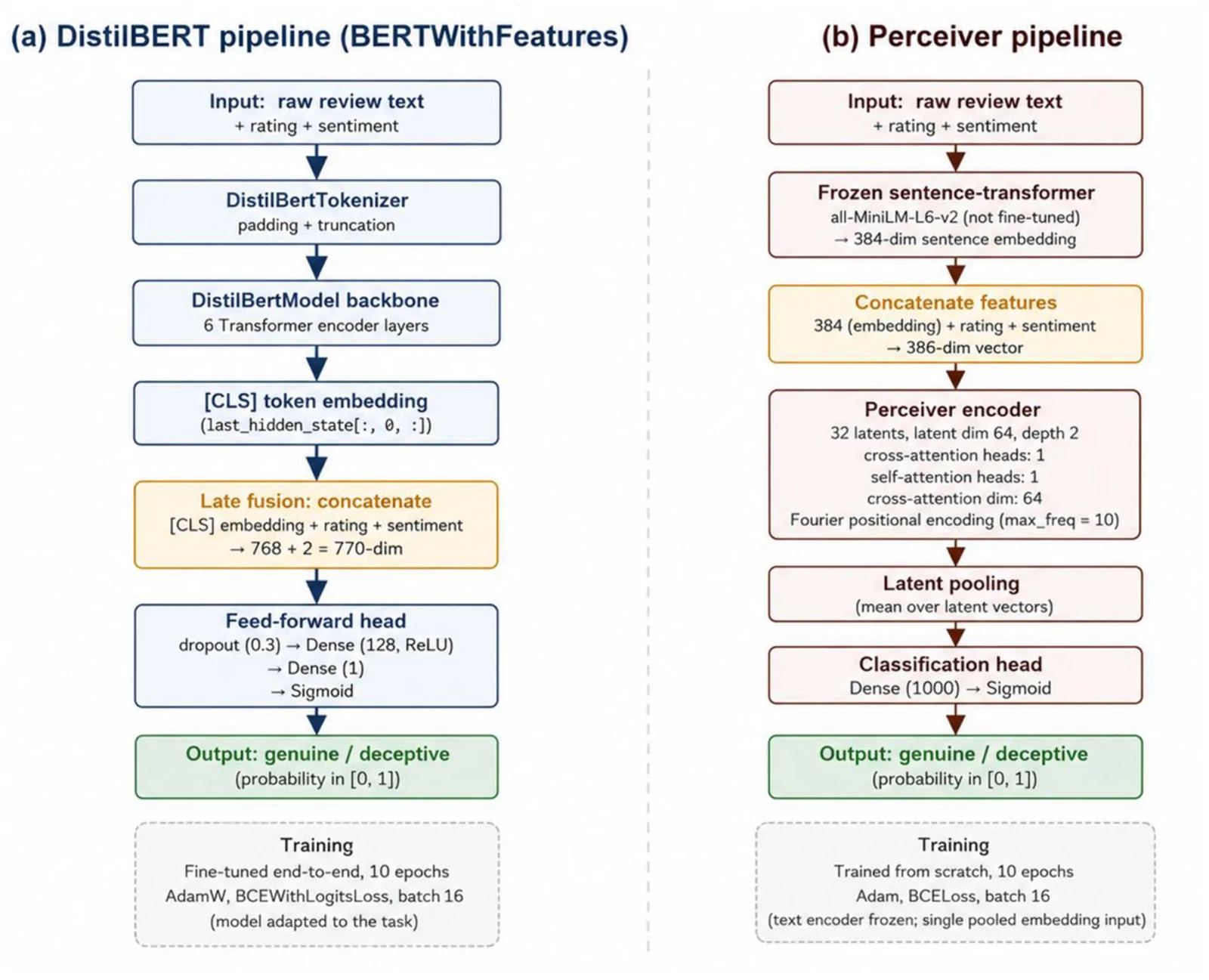
Logical flow of the two architectural pipelines. **(a)** DistilBERT pipeline (BERTWithFeatures); **(b)** Perceiver pipeline.

The logical flow of the two processing architectures is illustrated in Figure 1. As can be observed, the main difference is that DistilBERT adapts its text representation to the specific task, whereas the Perceiver operates on a fixed, task-agnostic text representation.

The comparison performed in this study should be interpreted as a comparison between two evaluated experimental pipelines rather than as a direct comparison of the intrinsic capabilities of the DistilBERT and Perceiver architectures.

To provide additional context for the performance of the proposed pipelines, several classical baseline models were also evaluated. These included a Dummy Classifier predicting the majority class, a TF-IDF representation combined with Logistic Regression, and a class-balanced TF-IDF with Logistic Regression. All baseline models were trained and evaluated using the same three stratified train–test splits as the deep learning pipelines to ensure a fair comparison. Their results are presented in Section 6.4.

## 5 Comparative performance analysis

This section presents a comparative evaluation of DistilBERT and Perceiver using accuracy, precision, recall, F1-score, log loss, and ROC-AUC. Results are discussed globally and per metric, with graphical representations used to highlight learning dynamics across the selected training epochs.

### 5.1 Accuracy

Accuracy expresses the proportion of correctly classified instances out of the total number of evaluated samples, providing an initial indication of how effectively a model distinguishes between truthful and deceptive reviews. Accuracy is a dimensionless performance metric bounded between 0 and 1, where higher values indicate a larger proportion of correctly classified instances and therefore better classification performance.

**Figure 2:**
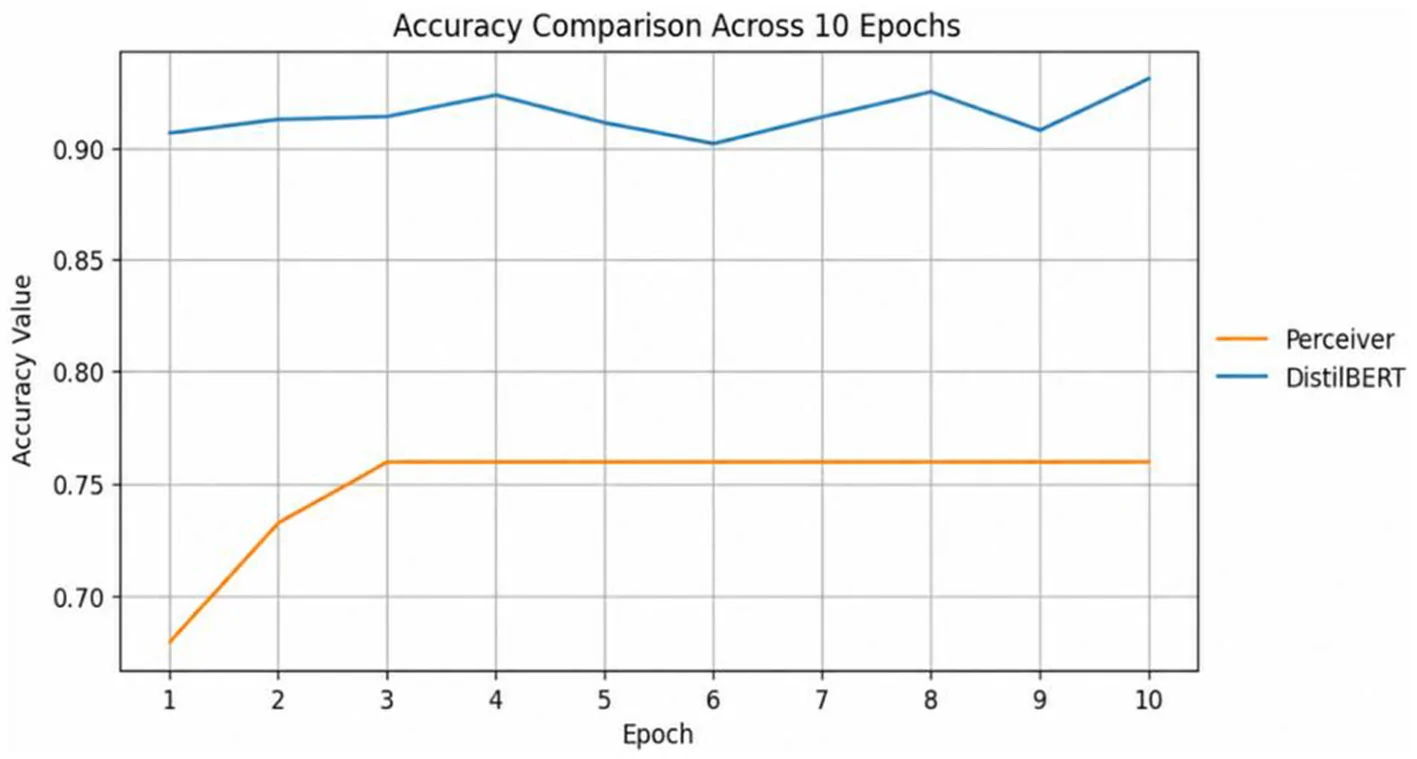
Accuracy evolution of DistilBERT and Perceiver over 10 training epochs.

**Figure 3:**
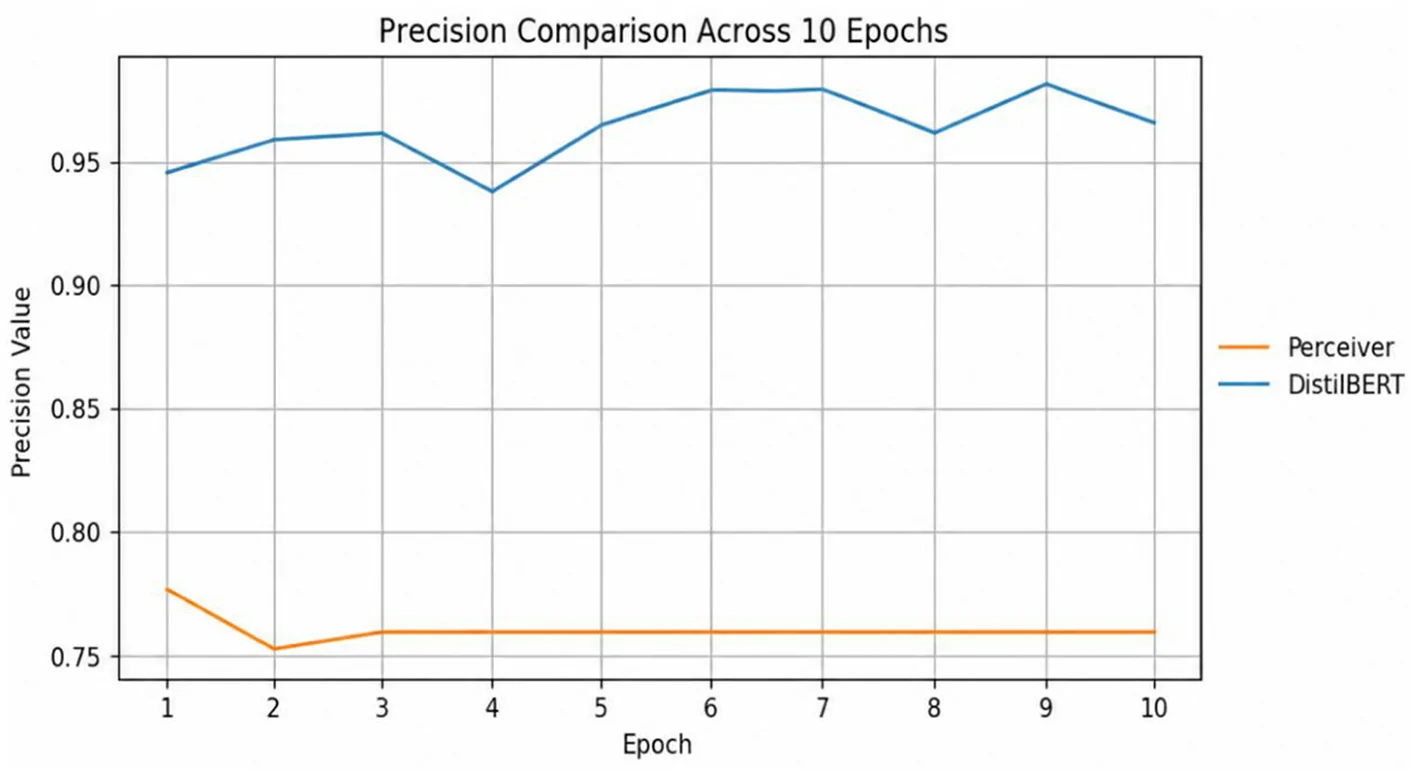
Precision evolution of DistilBERT and Perceiver over 10 training epochs.

**Figure 4:**
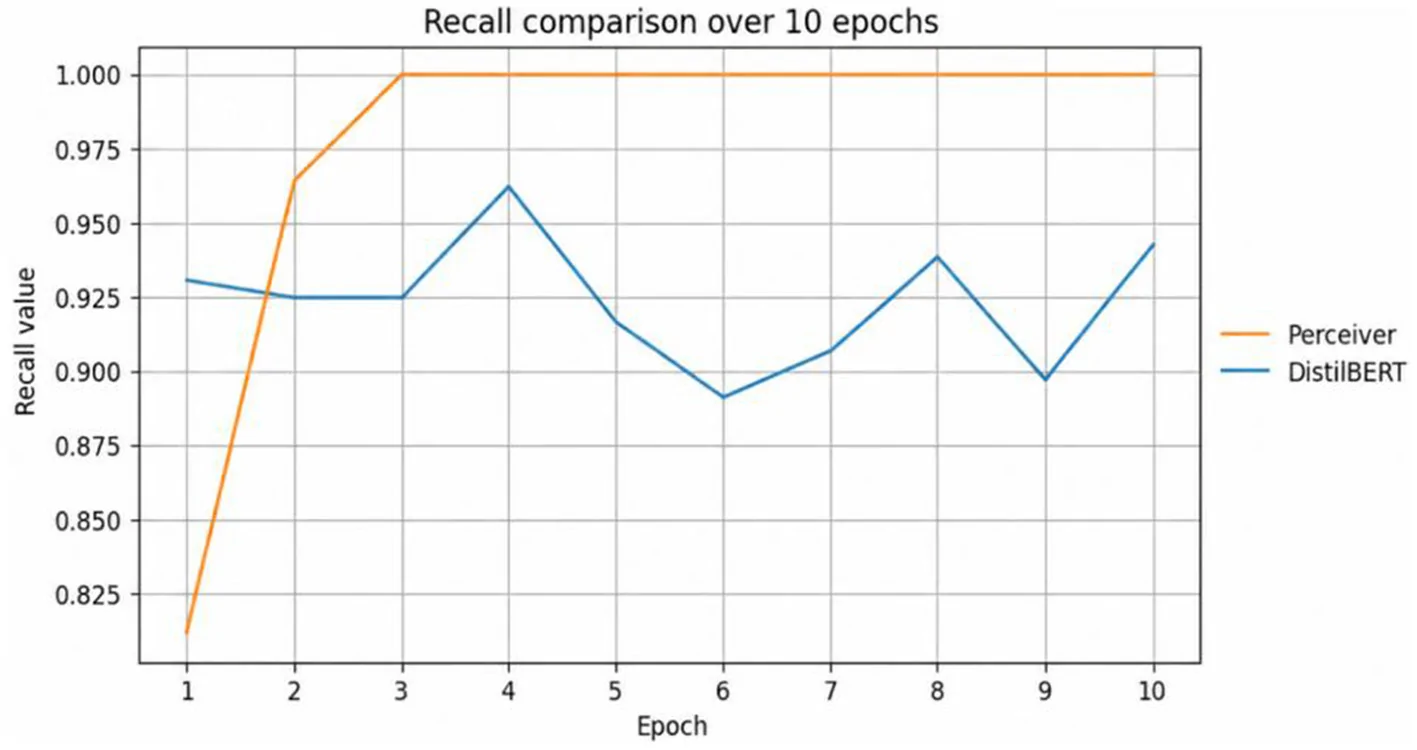
Recall evolution of DistilBERT and Perceiver over 10 training epochs.

**Figure 5:**
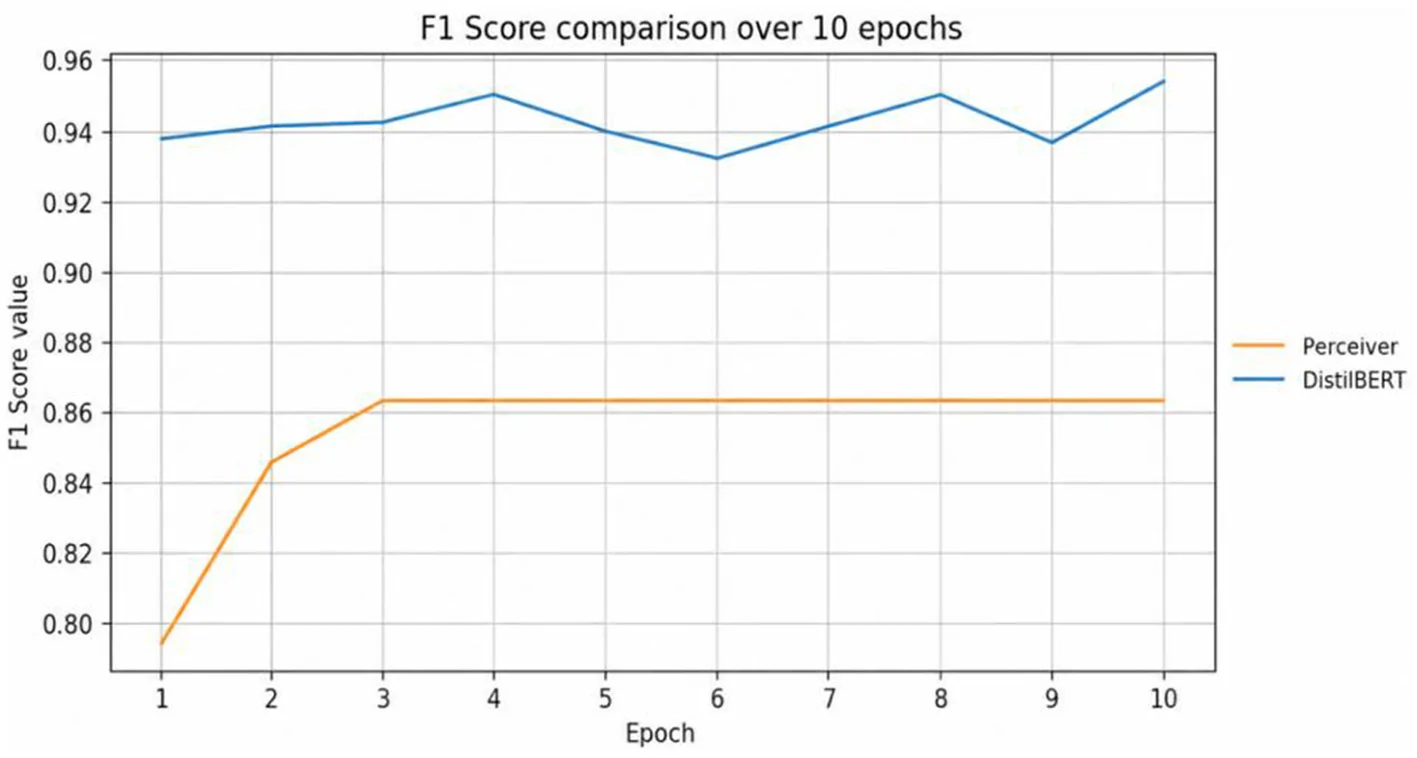
F1-score evolution of DistilBERT and Perceiver over 10 training epochs.

**Figure 6:**
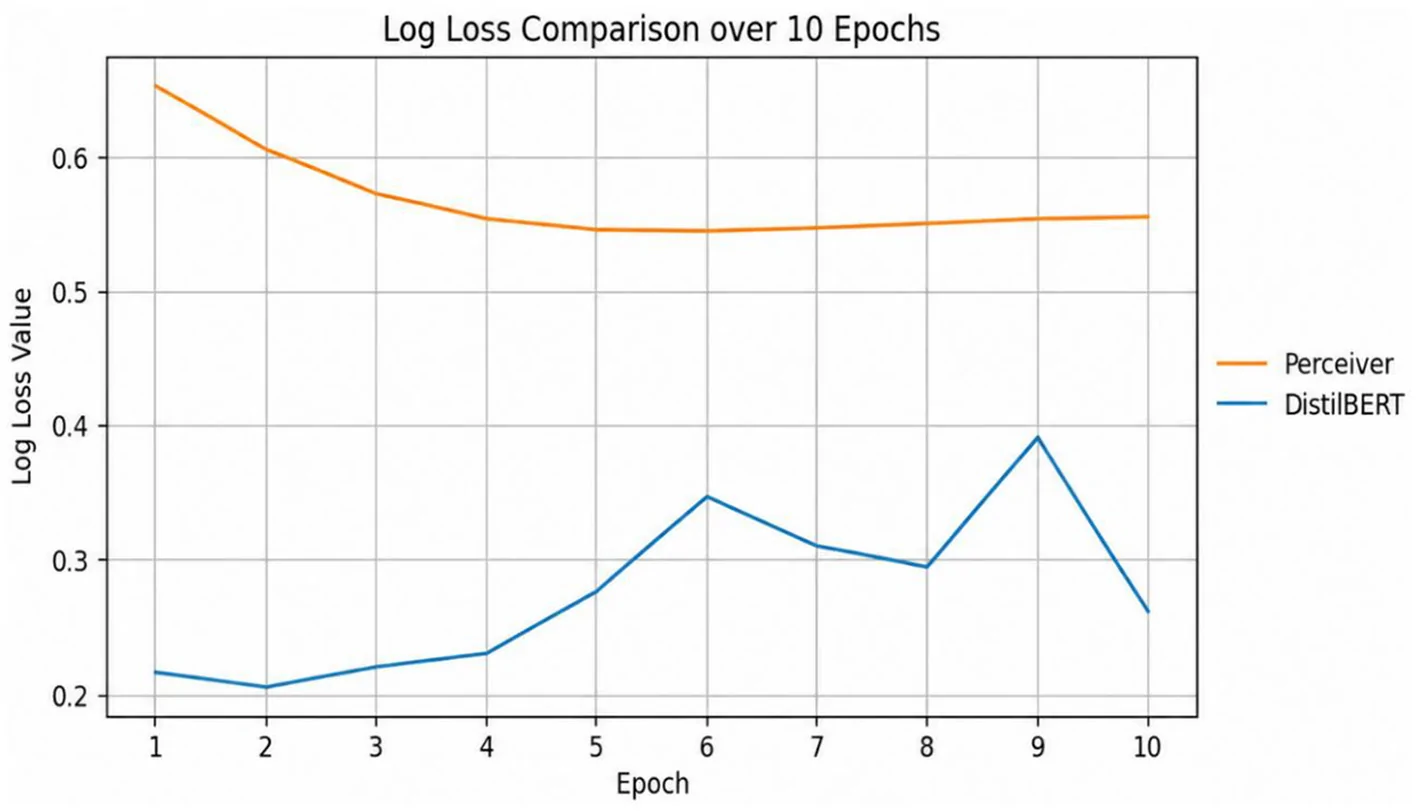
Log-loss evolution of DistilBERT and Perceiver over 10 training epochs.

**Figure 7:**
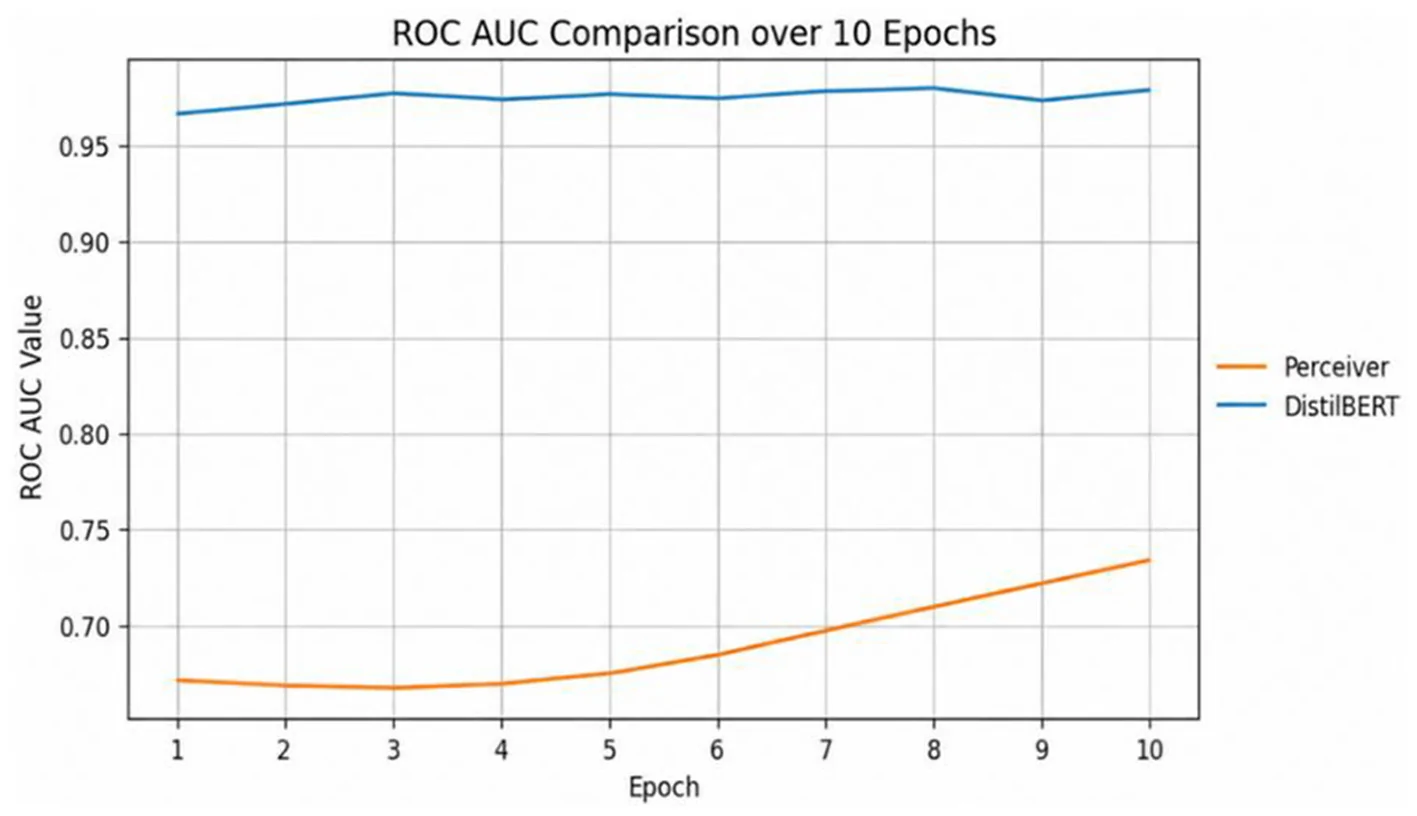
ROC-AUC evolution of DistilBERT and Perceiver over 10 training epochs.

Both models were trained for the full 10 epochs, and the corresponding metric curves over all 10 epochs are shown in the evolution figures (Figures 2–7). For the tabular comparison, however, three representative checkpoints–epochs 3, 5, and 10–were selected in order to keep the tables concise while still capturing the three qualitatively distinct phases of training: the early-convergence phase (epoch 3), an intermediate-adaptation phase (epoch 5), and the final-optimization phase (epoch 10). These three checkpoints were sufficient to characterize the behavior of both models, since, as the full-epoch figures confirm, the metrics either stabilize early (DistilBERT) or remain essentially flat across epochs (Perceiver); reporting every intermediate epoch in the tables would therefore add length without adding information.

As shown in Table 2, DistilBERT consistently outperforms Perceiver across all selected epochs. At epoch 3, DistilBERT reaches an accuracy of 0.9143, substantially exceeding the 0.7594 obtained by Perceiver. By epoch 10, DistilBERT improves to 0.9308, whereas Perceiver remains unchanged at 0.7594, indicating an early performance plateau with no measurable gain across checkpoints. At epoch 10, DistilBERT exceeds Perceiver by 0.1714 absolute accuracy points, a considerable gap for a binary classification task.

**Table 2:** Accuracy comparison between DistilBERT and Perceiver at representative training epochs.

Model	Epoch 3	Epoch 5	Epoch 10
DistilBERT	0.9143	0.9113	0.9308
Perceiver	0.7594	0.7594	0.7594

Figure 2 provides a broader view of model behavior across the full training process. DistilBERT maintains a high accuracy level throughout the 10 epochs, with minor fluctuations followed by a final increase, suggesting stable learning and progressive refinement of the classification boundary. By contrast, Perceiver shows only limited initial improvement and then converges to a nearly flat trajectory, indicating that additional epochs do not translate into better predictive performance.

### 5.2 Precision

Precision measures the proportion of correctly identified positive instances among all instances predicted as positive. In deceptive review detection, high precision indicates that the model reliably avoids labeling deceptive reviews as genuine.

Precision is a dimensionless performance metric bounded between 0 and 1, where higher values indicate that the model is more reliable when assigning the positive class and makes fewer false positive errors.

As shown in Table 3, DistilBERT consistently achieves substantially higher precision, reaching 0.9655 at epoch 10 compared with a static 0.7594 for Perceiver. The 0.2061 absolute difference at epoch 10 is practically significant: in hospitality review systems, falsely flagging genuine content as deceptive may affect business credibility and user trust.

**Table 3:** Precision comparison between DistilBERT and Perceiver at representative training epochs.

Model	Epoch 3	Epoch 5	Epoch 10
DistilBERT	0.9609	0.9646	0.9655
Perceiver	0.7594	0.7594	0.7594

Figure 3 provides a broader perspective on precision across the full training process. DistilBERT maintains a high level of precision over all epochs, with only minor fluctuations and a generally stable upward tendency. This behavior suggests robust learning and a sustained ability to make correct positive predictions throughout training. In contrast, Perceiver shows only limited variation in the early training phase and then converges to an almost flat trajectory, indicating that additional epochs do not meaningfully improve its precision performance.

### 5.3 Recall

Recall measures the proportion of actual positive instances correctly identified by the classifier—in this context, the fraction of truly genuine reviews that the model successfully identifies.

Recall is a dimensionless performance metric bounded between 0 and 1, where higher values indicate that a larger proportion of actual positive instances are correctly identified and fewer false negatives are produced.

Perceiver achieves a perfect recall of 1.0000 at all checkpoints, while DistilBERT records slightly lower but consistently high values (0.9168–0.9426) as can be seen in Table 4. However, recall must not be interpreted in isolation: as demonstrated below, Perceiver's perfect recall is an artifact of its collapsed classification strategy rather than a sign of genuine discriminative capability. DistilBERT maintains high recall while preserving a much stronger balance across all other metrics.

**Table 4:** Recall comparison between DistilBERT and Perceiver at representative training epochs.

Model	Epoch 3	Epoch 5	Epoch 10
DistilBERT	0.9248	0.9168	0.9426
Perceiver	1.0000	1.0000	1.0000

Figure 4 provides a more detailed overview of how recall changes throughout the training process. Perceiver reaches a recall value of 1.0 at an early stage and maintains this level across the remaining epochs, with no further variation observed. DistilBERT, in contrast, shows some fluctuations between epochs, although its recall remains consistently high throughout training. These results indicate different patterns of model behavior: Perceiver achieves stable recall relatively early, whereas DistilBERT continues to show small variations as training progresses. However, recall alone is not sufficient to determine whether one model adapts more effectively than the other, and these results should therefore be considered together with the other evaluation metrics.

### 5.4 F1-Score

The F1-score is the harmonic mean of precision and recall, providing a balanced measure of performance when both false positives and false negatives carry cost. It is a dimensionless performance metric bounded between 0 and 1, where higher values indicate a better balance between precision and recall and, consequently, superior overall classification performance.

As shown in Table 5, DistilBERT maintains a consistent advantage, reaching 0.9539 at epoch 10 against a static 0.8632 for Perceiver. The 0.0907 absolute difference reflects a meaningful gap in overall classification quality. DistilBERT's improving F1 trajectory suggests progressive learning across epochs, whereas Perceiver's flat curve indicates that additional training does not improve the precision–recall balance.

**Table 5:** F1-score comparison between DistilBERT and Perceiver at representative training epochs.

Model	Epoch 3	Epoch 5	Epoch 10
DistilBERT	0.9425	0.9401	0.9539
Perceiver	0.8632	0.8632	0.8632

A broader perspective is provided by Figure 5, which illustrates the evolution of F1-score across the full training process. DistilBERT maintains a high and relatively stable trajectory, with only small fluctuations and a final improvement at epoch 10. Such behavior suggests robust learning and sustained classification quality over time. Perceiver, on the other hand, shows a brief increase in the early epochs and then quickly settles into a flat curve, indicating that additional training does not contribute to a better balance between precision and recall.

### 5.5 Log loss

Log loss evaluates the confidence of probabilistic predictions by penalizing incorrect classifications more strongly when made with high certainty. Lower values indicate better-calibrated predictions. It is a dimensionless performance metric with a lower bound of 0 and no fixed upper bound, where smaller values indicate better calibrated probability estimates and more reliable predictions.

DistilBERT records markedly lower log-loss values at all checkpoints, remaining below 0.28 throughout training. Perceiver consistently exceeds 0.54, indicating weaker confidence calibration and less reliable probabilistic predictions. The 0.2920 absolute gap at epoch 10 underscores the greater trustworthiness of DistilBERT's probability estimates for automated review screening.

Figure 6 extends the comparison over the complete training trajectory. DistilBERT shows moderate variation across epochs, but its values remain within a comparatively low range throughout training. This behavior suggests that the model preserves a generally stable level of confidence even when some fluctuations appear. Perceiver, in contrast, follows an almost flat curve at a substantially higher level, indicating that additional epochs do not significantly improve the quality of its probabilistic predictions.

### 5.6 ROC-AUC

ROC-AUC measures the ability of a classifier to separate classes across different decision thresholds. Higher values indicate stronger discriminative capacity between deceptive and genuine reviews.

DistilBERT maintains ROC-AUC scores close to 0.98 throughout training, reflecting robust and stable class separability. Perceiver shows gradual improvement (0.6682 to 0.7339) but remains in a substantially lower range. The 0.2445 absolute gap at epoch 10 confirms that DistilBERT provides a far more reliable basis for ranking and discriminating between deceptive and authentic reviews. It is relevant to note that ROC-AUC is a dimensionless performance metric bounded between 0 and 1, where higher values indicate better class separability and ranking performance.

A more detailed view emerges from Figure 7, which tracks ROC-AUC over the full 10-epoch training process. DistilBERT follows a highly stable trajectory, remaining above 0.95 throughout and showing only small fluctuations. This pattern suggests that the model preserves strong discriminative behavior from the early stages of training onward. Perceiver displays a mild upward tendency across epochs, but the overall curve remains in a much lower performance region, indicating that the additional training only partially improves its ability to distinguish between deceptive and genuine reviews.

### 5.7 Cross-metric comparison

A cross-metric comparison provides an integrated view of the relative performance of the two models.

**Figure 8:**
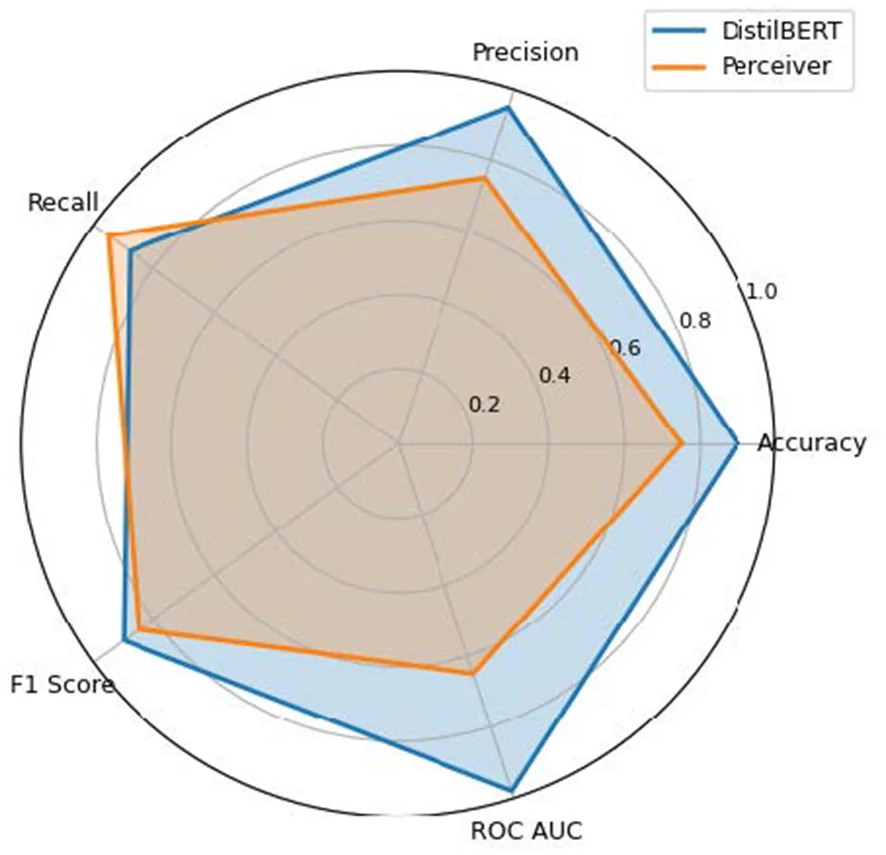
Cross-metric comparison of DistilBERT and Perceiver across the main evaluation metrics.

Figure 8 synthesizes the comparative results for the five-core metrics included in the global visual analysis, namely accuracy, precision, recall, F1-score, and ROC-AUC. This representation provides an immediate overview of the strengths and weaknesses of each model across the most relevant dimensions of classification performance.

DistilBERT achieves higher values in accuracy, precision, F1-score, and ROC-AUC, indicating better overall classification correctness, stronger prediction reliability, and superior class separability. Perceiver outperforms DistilBERT only in recall, where it reaches the maximum score—a result attributable to its systematic over-prediction of the positive class rather than to genuine discriminative capacity. The radar analysis confirms that DistilBERT maintains a more favorable performance distribution, whereas Perceiver exhibits an unbalanced profile with a single strong dimension and weak results across most other criteria.

## 6 Error analysis and confusion matrix interpretation

To complement the metric-based evaluation, an error-oriented analysis examines how the two models distribute their predictions across the target classes. Confusion matrices offer a more detailed perspective on classification decisions, particularly relevant for deceptive review detection where class-specific errors carry distinct practical consequences.

### 6.1 Confusion matrix analysis

**Figure 9:**
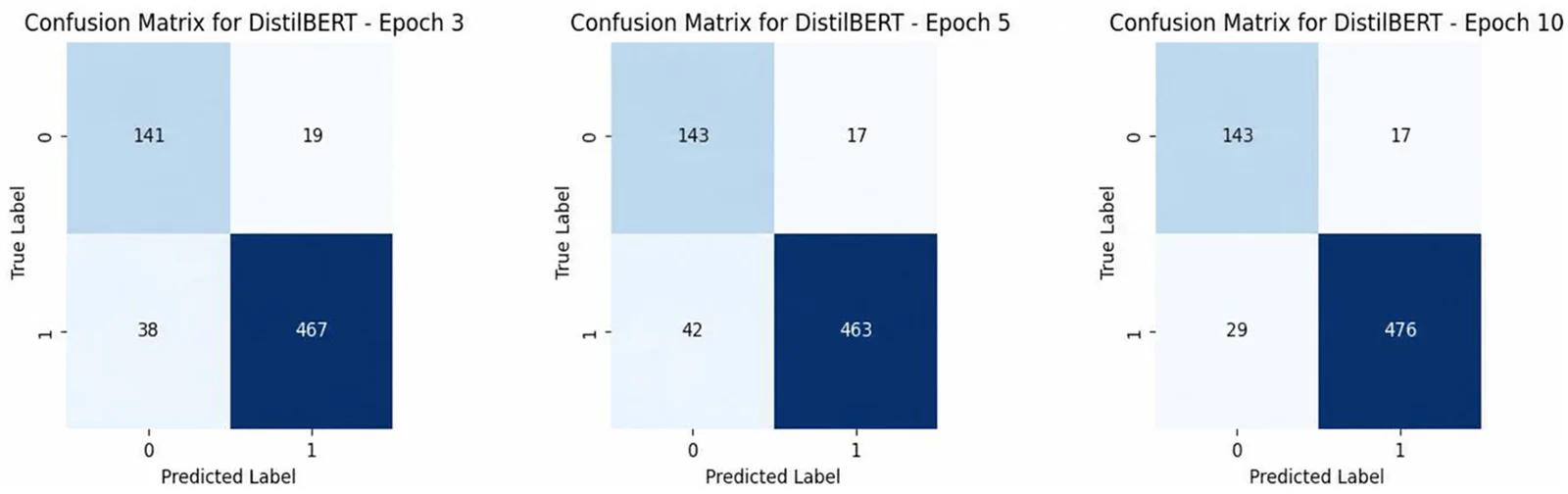
Confusion matrices for DistilBERT at epochs 3, 5, and 10.

The confusion matrices for DistilBERT (Figure 9) reveal a strong and relatively balanced classification pattern across all three representative epochs, with a high number of correct predictions for both classes and only a limited number of errors. Minor improvements are also observed across training, particularly by epoch 10, where the number of correctly identified instances increases and false negatives decrease.

**Figure 10:**
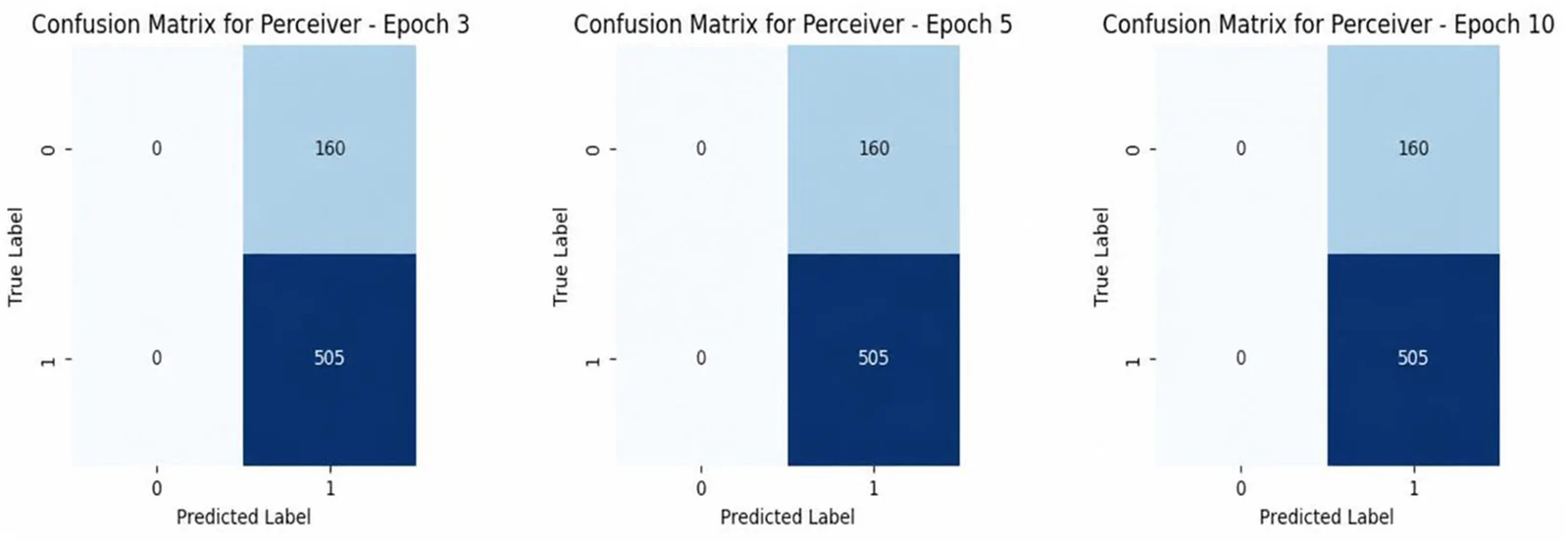
Confusion matrices for Perceiver at epochs 3, 5, and 10.

Perceiver shows a different pattern of results, as illustrated in Figure 10. At all three checkpoints, the model classifies nearly all instances as belonging to the positive class. As a result, genuine reviews are correctly identified, while deceptive reviews are rarely, if ever, detected. This explains the recall value of 1.0, but also reveals a substantial imbalance in the model's predictions. Rather than reflecting balanced classification performance, the high recall appears to result from a strong tendency to predict the majority class. This behavior may be related to the class distribution of the merged dataset, in which approximately 76% of the reviews are genuine and 24% are deceptive. Perceiver appears unable to learn a meaningful boundary between genuine and deceptive reviews under the current experimental conditions.

### 6.2 Comparison of correct and incorrect classification types

At epoch 10, the prediction breakdown on the 665-sample test set is as follows.

These figures clarify the trade-off suggested by the aggregate metrics. Perceiver correctly identifies slightly more genuine reviews (505 vs. 476 true positives) but produces an order of magnitude more false positives (160 vs. 17) and fails entirely to identify any deceptive review (0 true negatives vs. 143 for DistilBERT). DistilBERT delivers a far more balanced classification profile and is the only model that provides useful discrimination between the two classes. For practical review screening, a model that cannot distinguish genuine reviews from deceptive ones offers no actionable value, regardless of its recall score.

### 6.3 Imbalance-robust evaluation

Because accuracy, precision, and recall can be dominated by the majority class under a skewed class distribution, the two models are additionally evaluated with metrics that are robust to imbalance. Specificity, balanced accuracy, and the Matthews correlation coefficient (MCC) are computed directly from the epoch-10 confusion matrices reported in Section 6.1 and therefore introduce no additional modeling assumptions.

Table 6 makes the contrast unambiguous. DistilBERT retains high values across all imbalance-robust metrics, with a balanced accuracy of 0.9182 and an MCC of 0.8164, confirming genuine discrimination between the two classes. Perceiver, despite its perfect recall, attains a balanced accuracy of exactly 0.50–the value expected from a classifier that assigns every instance to the majority class–and an MCC of approximately 0, indicating no correlation between its predictions and the true labels. These results show that Perceiver's apparent strength on recall is an artifact of majority-class collapse rather than evidence of learning.

**Table 6:** Imbalance-robust metrics at epoch 10, computed from the confusion matrices in Section 6.1.

Metric (epoch 10)	DistilBERT	Perceiver
Recall (sensitivity)	0.9426	1.0000
Specificity	0.8938	0.0000
Balanced accuracy	0.9182	0.5000
Matthews correlation coefficient (MCC)	0.8164	0.0000^*^

^*^The MCC is undefined for Perceiver because the model predicts a single class (no true negatives and no false negatives, making one factor of the denominator zero); it is reported as 0 by the usual convention.

To further contextualize the Perceiver results, we additionally evaluated a binary Logistic Regression classifier as a linear baseline on the same frozen embeddings used as input for the Perceiver model. Logistic Regression was selected because it represents one of the simplest and most widely used linear classifiers for binary text classification and therefore provides an appropriate reference point for assessing whether the Perceiver's latent attention mechanism extracts information beyond that already encoded in the frozen sentence embeddings.

Specifically, we used the scikit-learn implementation of Logistic Regression with L2 regularization (penalty = “2”, *C* = 1.0), the lbfgs solver, and a maximum of 1,000 iterations. The model was trained on the same 386-dimensional input vectors (384-dimensional all-MiniLM-L6-v2 embeddings concatenated with rating and sentiment features) and evaluated on the same stratified 80/20 train-test split as the Perceiver model.

As shown in Table 1, Logistic Regression achieves performance very close to that of Perceiver, with only small differences in accuracy (0.7710 vs. 0.7594), F1-score (0.8700 vs. 0.8632), and balanced accuracy (0.5140 vs. 0.5000). These results suggest that Logistic Regression and Perceiver achieve broadly comparable performance on the same frozen embeddings and that the evaluated Perceiver configuration does not demonstrate a clear advantage over a simple linear baseline.

The results reported so far reflect the behavior of the two deep models without explicit imbalance mitigation. Section 6.4 therefore examines the effect of a class-weighted loss on Perceiver, together with baseline methods trained on the raw review text, and Section 6.5 reports an ablation of the auxiliary numeric features; all additional experiments are evaluated across three independent stratified splits. The remaining open question concerns Perceiver's behavior under further mitigation strategies, such as oversampling, under sampling, or decision-threshold calibration, which were not evaluated here; future work should examine whether these can further improve Perceiver's ability to recover meaningful discriminative capacity under imbalanced data conditions.

### 6.4 Baselines, imbalance mitigation and multi-split evaluation

At this point in the analysis, two aspects require closer consideration. The first concerns the interpretation of the performance scores themselves: an accuracy value above 0.93 is meaningful only when it is situated in relation to suitable reference models, including trivial classifiers and simpler text-based approaches trained on the same data. The second concerns the behavior of the Perceiver pipeline. More specifically, it is necessary to establish whether the collapse observed in its predictions should be attributed to limitations of the particular configuration evaluated here, or whether it is mainly a consequence of the class imbalance present in the corpus. The supplementary experiments introduced in Section 4.4 were therefore designed to clarify both issues. Tables 7, 8 report the results obtained across the three stratified train–test splits: Table 7 presents the standard classification metrics, whereas Table 8 provides imbalance-aware indicators and measures related to the calibration of the predicted probabilities.

**Table 7:** Conventional metrics across three stratified train–test splits (mean ± standard deviation).

Configuration	Accuracy	Precision	Recall	F1-score
Majority-class classifier	0.7594 ± 0.0000	0.7594 ± 0.0000	1.0000 ± 0.0000	0.8632 ± 0.0000
TF-IDF + logistic regression	0.8957 ± 0.0068	0.8842 ± 0.0064	0.9927 ± 0.0009	0.9353 ± 0.0040
TF-IDF + logistic regression (balanced)	0.9258 ± 0.0055	0.9777 ± 0.0057	0.9234 ± 0.0108	0.9497 ± 0.0040
Perceiver (as evaluated in Sections 5–6)	0.7594 ± 0.0000	0.7594 ± 0.0000	1.0000 ± 0.0000	0.8632 ± 0.0000
Perceiver + class-weighted loss	0.7278 ± 0.0201	0.8814 ± 0.0389	0.7439 ± 0.0115	0.8061 ± 0.0093
DistilBERT (text + rating + sentiment)	0.9348 ± 0.0014	0.9615 ± 0.0061	0.9525 ± 0.0086	0.9569 ± 0.0013

**Table 8:** Imbalance-robust metrics and probability calibration across the same three splits (mean ± standard deviation).

Configuration	Specificity	Balanced accuracy	MCC	ROC-AUC	Log loss
Majority-class classifier	0.0000 ± 0.0000	0.5000 ± 0.0000	0.0000 ± 0.0000	0.5000 ± 0.0000	8.6722 ± 0.0000
TF-IDF + logistic regression	0.5896 ± 0.0252	0.7912 ± 0.0131	0.7021 ± 0.0208	0.9791 ± 0.0006	0.2874 ± 0.0033
TF-IDF + logistic regression (balanced)	0.9333 ± 0.0179	0.9284 ± 0.0059	0.8137 ± 0.0111	0.9798 ± 0.0005	0.3174 ± 0.0077
Perceiver (as evaluated in Sections 5–6)	0.0000 ± 0.0000	0.5000 ± 0.0000	0.0000 ± 0.0000	0.7877 ± 0.0098	0.5394 ± 0.0111
Perceiver + class-weighted loss	0.6771 ± 0.1197	0.7105 ± 0.0541	0.3748 ± 0.0879	0.7837 ± 0.0204	0.6767 ± 0.0074
DistilBERT (text + rating + sentiment)	0.8792 ± 0.0212	0.9158 ± 0.0064	0.8240 ± 0.0012	0.9818 ± 0.0038	0.2516 ± 0.0372

The positive class is the majority (genuine) class; recall therefore measures the retrieval of genuine reviews, while the detection of deceptive reviews is captured by specificity in Table 8.

The majority-class classifier fixes the reference point: on this corpus, predicting “genuine” without inspecting the text yields an accuracy of 0.7594 and an F1-score of 0.8632. The unmodified Perceiver reproduces these values exactly, on every split, with the confusion matrix [[0, 160], [0, 505]] recurring unchanged; its specificity, balanced accuracy, and MCC are those of a classifier that never predicts the minority class. What distinguishes it from the trivial baseline is its ROC-AUC of 0.7877 ± 0.0098, appreciably above chance, which indicates that the frozen embeddings retain some information capable of ranking genuine above deceptive reviews even though the learned decision threshold does not exploit it.

Class weighting confirms that this ranking information can be recovered, if only partially. With a positive-class weight of approximately 3.15, specificity rises from 0.0000 to 0.6771 ± 0.1197, balanced accuracy from 0.5000 to 0.7105 ± 0.0541, and the MCC from 0.0000 to 0.3748 ± 0.0879: the model now identifies a majority of the deceptive reviews. The correction is nonetheless incomplete and unstable. Accuracy falls slightly to 0.7278 ± 0.0201, the log loss rises to 0.6767 ± 0.0074, and the standard deviation of specificity approaches 0.12, meaning that the behavior of the weighted model varies substantially from one split to the next. The collapse documented in Sections 6.1–6.3 is therefore not an artifact of omitting a standard imbalance fix; applying one lifts the model above degeneracy but leaves it well below every text-based alternative, which is consistent with the interpretation advanced in Section 4.5 that the frozen sentence embedding, rather than the imbalance alone, constrains what this pipeline can learn.

The classical baselines answer the first question, and their answer is more sobering than the original comparison suggested. TF-IDF with logistic regression, trained on the raw text and without class weighting, already reaches an accuracy of 0.8957 ± 0.0068 and an MCC of 0.7021 ± 0.0208, though its specificity of 0.5896 ± 0.0252 reveals a residual bias toward the majority class. Adding balanced class weights largely removes that bias: specificity rises to 0.9333 ± 0.0179 and the MCC to 0.8137 ± 0.0111, with an accuracy of 0.9258 ± 0.0055. The full DistilBERT model remains ahead, at 0.9348 ± 0.0014 accuracy and 0.8240 ± 0.0012 MCC, but the margin is roughly one percentage point of accuracy and one hundredth of an MCC point. On this corpus, therefore, the fine-tuned Transformer does not achieve a qualitatively different level of performance from a well-configured lexical model; its advantages are a better calibrated probability estimate (log loss 0.2516 ± 0.0372 against 0.3174 ± 0.0077) and a markedly greater consistency across splits, the standard deviation of its accuracy being roughly a quarter of that of the balanced baseline.

### 6.5 Ablation of the auxiliary numeric features

In addition to the review text, the evaluated models incorporate two auxiliary numerical features: the original rating score and a binary sentiment indicator. To assess the extent to which these features contribute to the final predictions, an ablation experiment was conducted with DistilBERT using only the review text as input, while keeping the remaining components of the training and evaluation pipeline unchanged. The text-only model was evaluated on the same three stratified splits as the full configuration, allowing a direct comparison between the two settings. The results of this analysis are presented in Table 9.

**Table 9:** Contribution of the rating and sentiment features to DistilBERT (mean ± standard deviation across three splits).

Metric	Text + rating + sentiment	Text only
Accuracy	0.9348 ± 0.0014	0.9223 ± 0.0262
Precision	0.9615 ± 0.0061	0.9720 ± 0.0160
Recall	0.9525 ± 0.0086	0.9254 ± 0.0505
F1-score	0.9569 ± 0.0013	0.9470 ± 0.0198
Specificity	0.8792 ± 0.0212	0.9125 ± 0.0538
Balanced accuracy	0.9158 ± 0.0064	0.9190 ± 0.0079
MCC	0.8240 ± 0.0012	0.8099 ± 0.0432
ROC-AUC	0.9818 ± 0.0038	0.9823 ± 0.0040
Log loss	0.2516 ± 0.0372	0.3023 ± 0.1159

The text-only configuration performs comparably to the full DistilBERT model. Removing the rating and sentiment features reduces accuracy by approximately 1.3 percentage points and lowers the MCC by 0.014. At the same time, mean precision, specificity, balanced accuracy, and ROC-AUC are marginally higher in the text-only setting, although these differences remain within the variation observed across the three stratified splits. The clearest difference between the two configurations concerns stability. The full model shows substantially smaller standard deviations, particularly for accuracy, where the value is 0.0014 compared with 0.0262, and for log loss, where it is 0.0372 compared with 0.1159. These results indicate that the auxiliary numerical features contribute limited discriminative information on their own. The main predictive signal comes primarily from the review text, while rating and sentiment appear to stabilize the model by reducing its sensitivity to the particular train–test partition used during training. Their role is therefore modest and secondary, and they are best understood as additional numerical features appended to the textual representation.

## 7 Confidence, probability, and review attribute analysis

### 7.1 Probability score distribution and confidence evolution

Based on the sigmoid probability outputs generated on the 665-sample test set, DistilBERT assigns probabilities that are more concentrated toward the extremes of the [0, 1] interval, whereas Perceiver produces scores distributed within a narrower range. This observation is consistent with the quantitative evaluation results reported previously: DistilBERT achieves a substantially higher ROC-AUC (0.9784 vs. 0.7339 at epoch 10, Table 10) and a lower log loss (0.2634 vs. 0.5554, Table 11), suggesting better separation between the two classes and more reliable probabilistic predictions.

**Table 10:** ROC-AUC comparison between DistilBERT and Perceiver at representative training epochs.

Model	Epoch 3	Epoch 5	Epoch 10
DistilBERT	0.9766	0.9760	0.9784
Perceiver	0.6682	0.6755	0.7339

**Table 11:** Log-loss comparison between DistilBERT and Perceiver at representative training epochs.

Model	Epoch 3	Epoch 5	Epoch 10
DistilBERT	0.2207	0.2772	0.2634
Perceiver	0.5740	0.5469	0.5554

The evolution of the average predicted probabilities across the 10 training epochs further illustrates this difference. For DistilBERT, the mean probabilities associated with the two classes remain clearly separated throughout training, indicating persistent discrimination between genuine and deceptive reviews. In contrast, the average probabilities produced by Perceiver remain much closer over all epochs, suggesting only limited improvement in class separability despite additional optimization steps.

These observations are derived from the sigmoid probability outputs produced by both models on the held-out test set of 665 reviews. For each epoch, the predicted probabilities were grouped according to the true class labels and their average values were analyzed. The resulting patterns are consistent with the quantitative metrics previously reported: DistilBERT achieves a lower log loss (0.2634 vs. 0.5554 at epoch 10, Table 11), indicating more reliable probabilistic predictions than Perceiver, and a substantially higher ROC-AUC (0.9784 vs. 0.7339, Table 10), indicating stronger separability between the score distributions of the two classes. Conversely, Perceiver's tendency to collapse toward the majority class (Table 12, Section 6.1) explains the narrower and less separated probability distributions observed in this analysis.

**Table 12:** Prediction categories at epoch 10 (test set, *n* = 665).

Category	DistilBERT	Perceiver	Difference
True positives	476	505	−29
True negatives	143	0	+143
False positives	17	160	−143
False negatives	29	0	+29

### 7.2 Probability behavior across sentiment

For DistilBERT, the predicted probabilities exhibit a broader distribution across positive and negative reviews, with observable differences between the two sentiment categories. This behavior suggests that the model's output probabilities vary according to the sentiment information present in the reviews. In contrast, Perceiver produces more compressed sentiment-specific distributions, with most probability values concentrated within a narrower interval, indicating weaker differentiation between the positive and negative sentiment groups.

**Figure 11:**
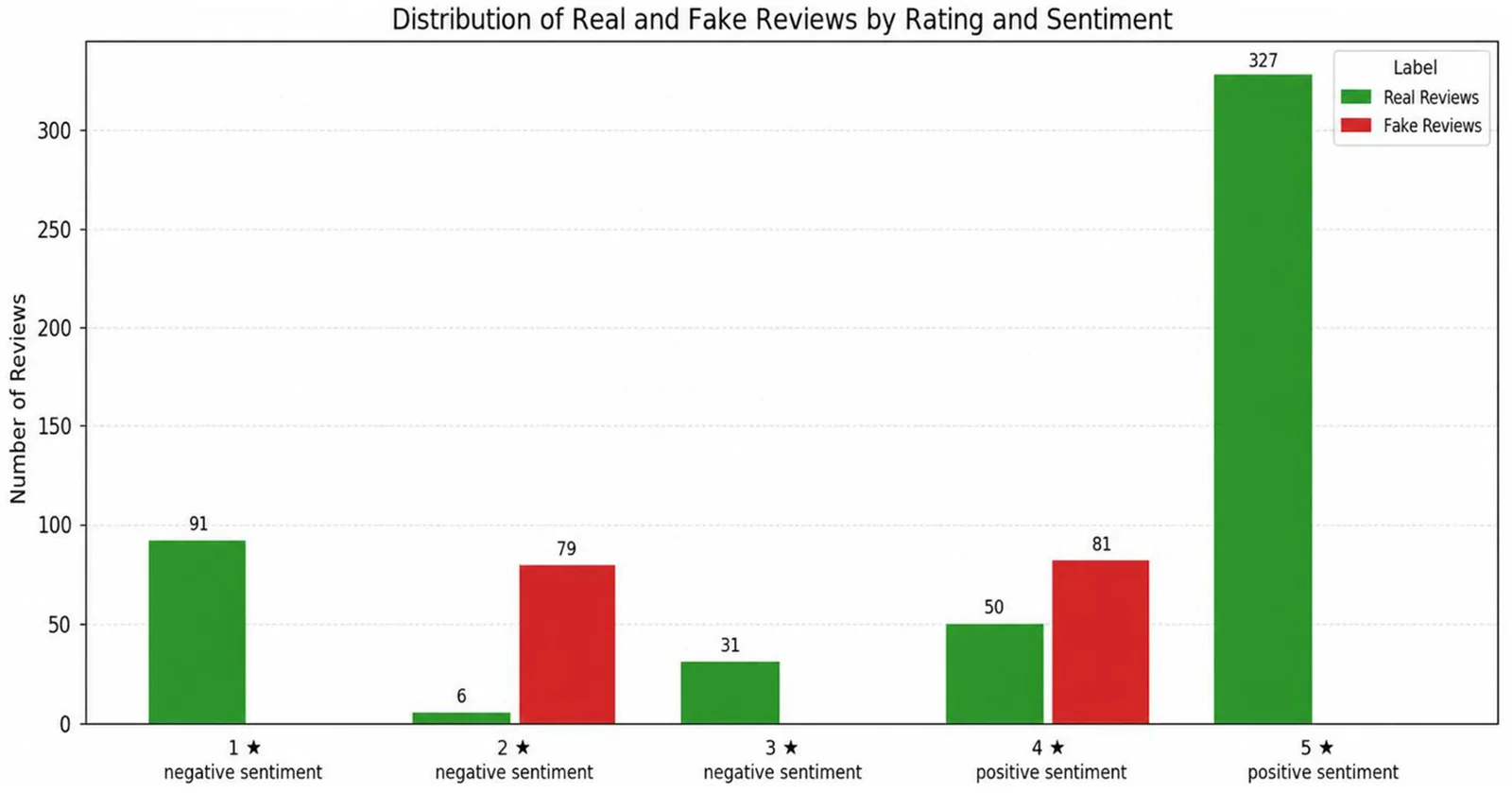
Distribution of genuine and deceptive reviews by rating and sentiment.

This comparison was obtained by grouping the predicted probabilities produced for each review in the 665-sample test set according to the binary sentiment indicator (positive vs. negative), derived as described in Section 4.2, and by comparing the resulting score distributions for each model. The relationship between sentiment, rating, and class membership underlying these groupings is illustrated in Figure 11.

The observed behavior is consistent with the quantitative results reported previously. DistilBERT's lower log loss (Table 11) and higher ROC-AUC (Table 10) indicate more reliable and better-separated probability estimates, whereas Perceiver's collapse toward the majority class (Section 6.1) explains the narrower probability distributions and the limited variation across sentiment categories observed here.

### 7.3 Performance across review attributes

**Figure 12:**
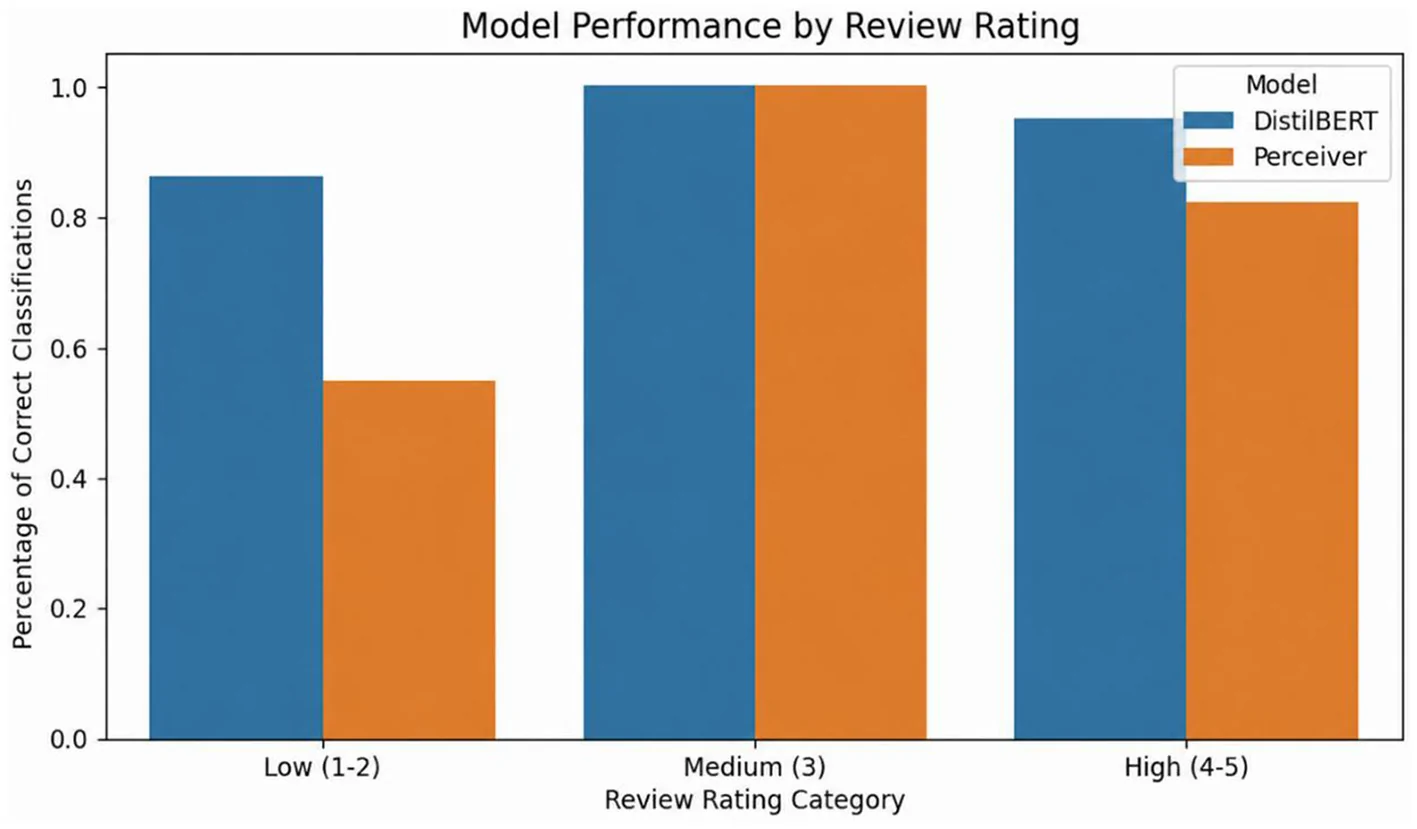
Model performance by review rating category.

Performance differences also become visible when the reviews are grouped by rating. As illustrated in Figure 12, DistilBERT maintains stronger and more consistent results across the rating categories, particularly for low and high ratings. Perceiver performs comparably only in the middle rating range, while its results decline more noticeably for strongly polarized reviews. These results suggest that DistilBERT maintains more stable predictive performance across the different rating categories, whereas Perceiver exhibits larger performance variations depending on the rating group considered.

In Figure 12, the reviews are grouped into three rating bands according to their original 1–5 star rating scale. The Low category includes reviews rated 1 or 2 stars, the Medium category includes reviews rated 3 stars, and the High category includes reviews rated 4 or 5 stars. This aggregation was introduced to analyze whether model performance remains stable across reviews expressing different levels of user satisfaction and rating intensity. The Low and High groups represent the two extremes of the rating scale, whereas the Medium group corresponds to intermediate ratings that may contain more neutral or mixed opinions. Grouping the reviews into these three broader categories also increases the number of samples in each subgroup, enabling a more reliable comparison of model behavior across rating levels.

Figure 11 shows the distribution of genuine and deceptive reviews across different combinations of ratings and sentiment. The proportions of the two classes vary across these categories, with some showing a relatively balanced distribution and others being dominated by one class. Reviews with highly positive sentiment are more frequently associated with the genuine class, whereas deceptive reviews account for a larger proportion of some negative and moderately negative categories. These differences suggest that rating and sentiment may also be associated with the class distribution. Therefore, when interpreting the classification results, it is useful to consider these characteristics alongside the textual information used by the models.

The distribution of genuine and deceptive reviews across rating and sentiment combinations reveals that highly positive reviews are pre-dominantly genuine, while certain negative or moderately negative categories show a larger deceptive component—a pattern that DistilBERT generalizes across more effectively.

## 8 Overall comparative discussion and model-wise strengths and limitations

The comparative evaluation conducted in this study highlights a clear difference between the two investigated models, DistilBERT and Perceiver, in the task of deceptive review detection. When the results are considered as a whole, the evaluated DistilBERT pipeline consistently achieved higher performance than the evaluated Perceiver pipeline across the main evaluation metrics and maintained a more balanced classification profile throughout training.

For DistilBERT, the main advantage lies in its superior results on the most relevant performance indicators, including accuracy, precision, F1-score, and ROC-AUC, regardless of the selected epoch. This behavior suggests that, under the evaluated configuration, the DistilBERT pipeline was able to learn and generalize more effectively from the available data. Another important strength is its stability over time, since the model preserves high and relatively constant results from epoch 3 onward, indicating good convergence during training. In addition, DistilBERT shows a more balanced classification behavior, with high values for both precision and recall, which supports a better compromise between correctly identifying deceptive reviews and avoiding excessive classification errors. Its strong ROC-based results further indicate an effective capacity to separate the two classes, while the lower log-loss values suggest greater confidence and better calibration in prediction.

At the same time, DistilBERT also presents certain limitations. Compared with Perceiver, it is computationally more demanding, due to its transformer-based architecture, and requires more resources for both training and inference. Its training time is also higher, which may be relevant in scenarios where computational efficiency is a critical constraint.

By contrast, the evaluated Perceiver pipeline employs a simpler model configuration, which may facilitate faster implementation and make it more attractive in settings with limited computational resources. Another notable characteristic is its constant recall of 1.000, which indicates that the model does not miss positive instances in the evaluated setting.

However, the evaluated Perceiver pipeline also exhibits several important limitations under the adopted experimental configuration. Its overall performance remains clearly below that of DistilBERT, with significantly lower values for accuracy, precision, F1-score, and ROC-AUC. In addition, the metric values remain almost unchanged or evolve only slightly across epochs, indicating limited adaptability and weak learning progression. A further drawback is the imbalance between metrics: the combination of very low precision and perfect recall suggests that the model tends to overpredict a single class, which negatively affects the overall quality of classification. This interpretation is also supported by the higher log-loss values, which indicate weaker confidence and poorer calibration of the predicted outputs.

Accordingly, the Perceiver results should not be generalized beyond the experimental conditions examined in this study. In the evaluated configuration, the model showed a limited and unstable ability to distinguish between genuine and deceptive reviews, but this should not be read as evidence that the Perceiver architecture is inherently unsuitable for this task. Rather, the results reflect the constraints of the specific pipeline, in which Perceiver operated on frozen sentence embeddings rather than task-adapted token-level representations. The baseline experiments further clarify the comparison: the original Perceiver matched the majority-class classifier in accuracy, indicating majority-class collapse, while class-weighted training partially improved its discriminative ability without reaching the performance of DistilBERT or the strongest TF-IDF plus logistic regression baseline. DistilBERT consistently achieved the most balanced and stable results, although its advantage over the balanced lexical baseline was modest and appeared mainly in probability calibration and cross-split stability. Rather than establishing a definitive hierarchy between DistilBERT and Perceiver as architectures, the present results highlight how different pipeline designs influence model behavior in deceptive review detection. This comparison offers a constructive foundation for future research, particularly through validation on larger and more diverse datasets, the exploration of alternative Perceiver input representations, and the inclusion of additional Transformer-based baselines such as BERT and RoBERTa.

## 9 Conclusions

This study presented a comparative empirical evaluation of DistilBERT and Perceiver for the binary classification of genuine vs. deceptive hotel reviews, using a combined corpus of 3,322 English-language reviews drawn from the Myleott benchmark corpus (Ott et al., 2011) and a TripAdvisor dataset of Ritz-Carlton reviews sourced from Kaggle (2024). The experimental results consistently show that the evaluated DistilBERT pipeline achieved higher and more balanced performance than the evaluated Perceiver pipeline across the considered evaluation metrics, while maintaining stable generalization throughout training under the adopted experimental configuration.

The repeated evaluation across three stratified random splits confirmed that these findings were not dependent on a single train–test partition. The full DistilBERT pipeline achieved a mean accuracy of 0.9348 ± 0.0014, an F1-score of 0.9569 ± 0.0013, and an MCC of 0.8240 ± 0.0012, demonstrating both strong performance and low cross-split variability. In contrast, the original Perceiver pipeline reproduced the majority-class collapse in all three runs. Class-weighted loss improved its ability to distinguish the minority class, but the resulting balanced accuracy and MCC remained substantially lower and more variable than those of DistilBERT and the strongest classical baseline.

Although Perceiver proved computationally efficient and achieved perfect recall, its predictions revealed a collapsed classification pattern rather than a meaningful separation between genuine and deceptive reviews. Specifically, the model assigned all test instances to the positive class, thereby reproducing the majority-class tendency instead of learning a discriminative decision boundary. This outcome can be explained, at least partly, by the class imbalance of the merged dataset, in which genuine reviews represented approximately 76% of the samples and deceptive reviews approximately 24%. Training the model with a class-weighted loss prevented the complete collapse and restored partial discriminative capacity (balanced accuracy 0.7105 ± 0.0541, MCC 0.3748 ± 0.0879), yet performance remained well below both DistilBERT and the classical baselines and varied considerably across splits, indicating that the difficulty is not resolved by a standard imbalance correction alone. A second factor is the asymmetry between the two pipelines: DistilBERT adapts its word representations to the task, whereas Perceiver receives a fixed embedding from a frozen encoder and cannot modify the textual representation it is given. Consequently, the findings emphasize the need to report class distributions explicitly and to address imbalance when designing, evaluating, and interpreting deceptive review detection experiments.

Both models incorporated two auxiliary structured features–the numerical rating and a binary sentiment indicator–in addition to the review text; these are best described simply as extra numeric features appended to the textual representation (Section 4.5). In the DistilBERT architecture, these features were concatenated with the pooled [CLS] representation before the classification head, following a late-fusion strategy. In the Perceiver configuration, they were appended to the frozen sentence embedding used as model input. The ablation reported in Section 6.5 shows their individual contribution to be modest: removing them costs about 1.3 percentage points of accuracy, while their clearest effect is a marked reduction in the variability of the results across splits. The predictive signal thus originates primarily from the review text itself, with the numeric attributes acting as a stabilizing complement rather than as an independent source of discriminative information.

Within the evaluated setting, the outcomes indicate that a pre-trained Transformer such as DistilBERT provides an effective basis for the automatic detection of deceptive reviews and identify the evaluated DistilBERT pipeline as the stronger of the two pipelines under the adopted experimental conditions. However, given the limited size of the corpus and its provenance from two English-language sources covering a small number of hotels, the reported results should be viewed as an encouraging but context-specific baseline.

Future research could extend the present study in several directions. One possibility would be to evaluate Perceiver directly on token-ID sequences, allowing both models to process the same type of textual input and enabling a more direct comparison between their architectures. Further experiments could also include statistical significance testing, for example using McNemar's test for paired predictions, as well as confidence intervals calculated across a larger number of data splits. Evaluating the models on larger datasets covering multiple languages and domains would provide further evidence of their ability to generalize beyond the current experimental setting. Finally, future studies could examine whether additional information about reviewer activity and behavior contributes to more accurate and consistent detection of deceptive reviews.

## Data Availability

The original contributions presented in the study are included in the article/supplementary material, further inquiries can be directed to the corresponding authors.
